# Discovering Potential Taxonomic Biomarkers of Type 2 Diabetes From Human Gut Microbiota *via* Different Feature Selection Methods

**DOI:** 10.3389/fmicb.2021.628426

**Published:** 2021-08-25

**Authors:** Burcu Bakir-Gungor, Osman Bulut, Amhar Jabeer, O. Ufuk Nalbantoglu, Malik Yousef

**Affiliations:** ^1^Department of Computer Engineering, Faculty of Engineering, Abdullah Gül University, Kayseri, Turkey; ^2^Department of Computer Engineering, Genome and Stem Cell Center, Erciyes University, Kayseri, Turkey; ^3^Department of Information Systems, Zefat Academic College, Zefat, Israel; ^4^Galilee Digital Health Research Center, Zefat Academic College, Zefat, Israel

**Keywords:** feature selection, metagenomic analysis, classification, machine learning, type 2 diabetes, human gut microbiome

## Abstract

Human gut microbiota is a complex community of organisms including trillions of bacteria. While these microorganisms are considered as essential regulators of our immune system, some of them can cause several diseases. In recent years, next-generation sequencing technologies accelerated the discovery of human gut microbiota. In this respect, the use of machine learning techniques became popular to analyze disease-associated metagenomics datasets. Type 2 diabetes (T2D) is a chronic disease and affects millions of people around the world. Since the early diagnosis in T2D is important for effective treatment, there is an utmost need to develop a classification technique that can accelerate T2D diagnosis. In this study, using T2D-associated metagenomics data, we aim to develop a classification model to facilitate T2D diagnosis and to discover T2D-associated biomarkers. The sequencing data of T2D patients and healthy individuals were taken from a metagenome-wide association study and categorized into disease states. The sequencing reads were assigned to taxa, and the identified species are used to train and test our model. To deal with the high dimensionality of features, we applied robust feature selection algorithms such as Conditional Mutual Information Maximization, Maximum Relevance and Minimum Redundancy, Correlation Based Feature Selection, and select K best approach. To test the performance of the classification based on the features that are selected by different methods, we used random forest classifier with 100-fold Monte Carlo cross-validation. In our experiments, we observed that 15 commonly selected features have a considerable effect in terms of minimizing the microbiota used for the diagnosis of T2D and thus reducing the time and cost. When we perform biological validation of these identified species, we found that some of them are known as related to T2D development mechanisms and we identified additional species as potential biomarkers. Additionally, we attempted to find the subgroups of T2D patients using *k*-means clustering. In summary, this study utilizes several supervised and unsupervised machine learning algorithms to increase the diagnostic accuracy of T2D, investigates potential biomarkers of T2D, and finds out which subset of microbiota is more informative than other taxa by applying state-of-the art feature selection methods.

## Introduction

Trillions of living creatures live in our bodies, especially in our gut. These organisms are important to regulate our immune system. They provide energy, break down foreign matters, produce some hormones, etc., which are extremely important for our health. The gut microbiome including different types and amounts of microorganisms is crucial for human health and human disorders (Valdes et al., 2018). With the help of new technologies and methods, we can get gut microbiome data. In other words, we can measure their amount in our gut more easily than ever before. Hence, we can try to go after some correlation signs between these creatures and human diseases. Type 2 diabetes (T2D) is one of such diseases, which affects millions of people around the world. Approximately 9–11% of people in the United States and China have T2D. Four hundred sixty-three million people in the world, who are older than 20, have diabetes. One of three people in the United States, who are older than 20, has prediabetes. Seventy percent of these prediabetic individuals will also have diabetes (James et al., 2003; National Diabetes Clearinghouse, 2011; Tabak et al., 2012; Diabetes.co.uk, 2019; International Diabetes Federation, 2019; Centers for Disease Control and Prevention, 2020).

Several studies have been conducted on human microbiota and its relations with type 1 diabetes, T2D, or obesity (Turnbaugh et al., 2009; Vrieze et al., 2012; Trøseid et al., 2013; Boulangé et al., 2016; Chobot et al., 2018; Peters et al., 2018). Brunetti (2007) defined T2D as a worldwide epidemic in 2010 and claimed that obesity was one of the most important driving forces for the development of T2D. This is varied by ethnicity though. For North America, the relationship between T2D and obesity is 90%. Whereas it is smaller than 40% in South Asia (International Diabetes Federation, 2003; James et al., 2003). The microbiota studies for obesity is also important for T2D studies. Not all obese individuals have also T2D, but 86% of T2D individuals are obese or overweight (Daousi et al., 2006; Narayan et al., 2007). The diet is one of the important factors that affect the gut microbiota (Falony et al., 2016; Zhernakova et al., 2016). found that while the dietary changes have a 57% role for the gut microbiota variations, the genetic mutations only have 12% role. Despite that there are some contrary arguments, it is reported in Zhang et al. (2010) that we can slow down the increase of obesity, and so the T2D, by regulating the variations of our gut microbiota by doing dietary changes. After the meal, even the glycemic action type of a body can be affected by its gut microbiota composition (Zeevi et al., 2015; Mendes-Soares et al., 2019). Some studies show that biotin deficiency may be associated with T2D (Maebashi et al., 1993; Wu et al., 2020) and biotin supplementation may help glucose regulation (Fernandez-Mejia, 2005; Albarracin et al., 2008; Lazo de la Vega-Monroy et al., 2013).

Conducting different studies to discover the associations and the relationships between variations of the gut microbiota and T2D is essential. For example, Karlsson et al. (2013) emphasize the importance of gender, age, and family history in these kinds of studies. Therefore, in order to minimize the source of variation, they worked on such data that consist of 145 women who are 70 years old. Interestingly, they found that some *Lactobacillus* species are increased and some *Clostridium* species are decreased in the microbiomes of the T2D patients. They got 0.83 AUC with a metagenomics cluster level. Increased *Clostridium clostridioforme* and decreased *Roseburia* in T2D patients are common findings of Karlsson et al. (2013) and Qin et al. (2012). Larsen et al. (2010) and Lê et al. (2013) also found that *Lactobacillus* species are increased in T2D patients.

Forslund et al. (2015) presented a different perspective such that the possible effects of the T2D drugs on the human gut microbiome also need to be taken into account. They also addressed the need to disentangle microbiota signs of the disease from the medications that patients use. Forslund et al. (2015), Wu et al. (2017), and Sun et al. (2018) show the effects of the most commonly used anti-T2D drug metformin. But they also found that metformin-untreated T2D is still associated with the butyrate producer species deficiency. The importance of butyrate-producing species for glucose health is also emphasized by Karlsson et al. (2013), Qin et al. (2012), Allin et al. (2018), and Sanna et al. (2019). Wu et al. (2020) also showed that butyrate producers’ deficiency and the loss of genes for butyrate synthesis from both proteins and carbohydrates start to occur even from the prediabetic level. Diet is also important at this point, as mentioned before. The function of butyrate producers is also regulated by diet, especially fiber intake, which positively affects glucose control (Makki et al., 2018; Zhou et al., 2019).

Wu et al. (2020) also considered the potential effects of drugs on gut microbiota, and they studied the diabetes treatment-naive T2D cohort. Their findings were also in agreement with earlier studies (Qin et al., 2012; Karlsson et al., 2013; Forslund et al., 2015; Allin et al., 2018). They showed that their finding was independent of metformin, other confounding factors affecting gut microbiota, and also other confounders like age, BMI, and sex. Their microbiome-based machine learning model to detect T2D samples and healthy samples generated a 0.78 AUC score.

Zhong et al. (2019) worked on 254 samples of Chinese cohort. They found that *Dialister nvisus* (MLG-3376) and *Roseburia hominis* (MLG-14865 and 14920) are lower in the T2D patients who were also reported before by Forslund et al. (2015). They also found that *Streptococcus salivarius* (MLG-6991) is high in the pre-sick people, which is in agreement with the previous findings of Allin et al. (2018) in the Danish prediabetic cohort. Zhong et al. showed that *Megasphaera elsdenii* (MLG-1568) was found in higher amounts in T2D patients compared to the pre-DM and healthy individuals. A similar finding was previously presented by He et al. (2018) by conducting a study on 7,000 individuals from South China.

On the other hand, Thingholm et al. (2019) claim that we need to differentiate the gut microbiota of obese individuals with T2D and obese individuals without T2D. This is proposed because they show different functional capacities and composition. Obesity is more associated with alterations in microbiome composition than T2D. They also concluded that only nominal increases in *Escherichia/Shigella* happen in the microbiomes of T2D patients. Also, medications and dietary supplements are highly related to gut microbiome variations (Thingholm et al., 2019).

Another important point to consider is the daily changes of the microbiota. There are some studies about gut microbiota’s diurnal oscillations in composition (Thaiss et al., 2014; Liang et al., 2015; Kuang et al., 2019). More specifically to diabetes, Reitmeier et al. (2020) found that T2D patients exhibit disrupted circadian rhythms in their microbiome. They show that arrhythmic bacterial signatures have an additional value for the classification of T2D, and they found that 13 arrhythmic bacterial species contribute to risk profiling of T2D. On the other hand, they found that daily dietary habits (like mealtime or number of meals per day) are independent of gut microbiota composition (Reitmeier et al., 2020).

A recent survey paper by Marcos-Zambrano et al. (2021) summarized the applications of machine learning in the human microbiome studies and reviewed popular feature selection, biomarker identification, disease prediction, and treatment strategies. In this review, the most widely used machine learning algorithms that were used for microbiome analysis were reported as Random Forest, support vector machines (SVM), Logistic Regression, and k-NN. However, no clear recommendation is given and they have suggested to perform comparison study to choose the one with the optimal performance. All of those algorithms require a parameter tuning step to achieve its optimal model.

In this study, we analyzed T2D-associated metagenomic dataset *via* some feature selection algorithms such as Fleuret’s Conditional Mutual Information Maximization (CMIM), Peng’s Maximum Relevance and Minimum Redundancy (mRMR), Fast Correlation Based Filter (FCBF), and select K best (SKB). To assess the performance of different classifiers, in our preliminary analysis, we used Random Forest (RF), Decision Tree, Logitboost, Adaboost, SVM, and K-NN as classification methods. In our further experiments, we focused on RF classifier. In summary, this study utilizes both supervised and unsupervised machine learning algorithms (i) to generate a classification model that aids T2D diagnosis, (ii) to investigate potential pathobionts of T2D, and (iii) to find out subgroups of T2D patients.

The rest of this paper is organized as follows. In section “Materials and Methods”, we present the dataset that we have used in this study and we describe our methodology. In section “Experiments”, we present our findings when we apply feature selection algorithms, classification methods, and clustering algorithms to T2D-associated metagenomic data. In section “Discussions”, we discuss the identified species in our study as candidate taxonomic biomarkers of T2D and compare them with the gold standard features that are known to be associated with T2D in literature. In section “Conclusion”, we conclude the manuscript.

## Materials and Methods

In this study, we used the raw microbiome DNA sequencing data of 290 human samples. The raw sequencing data of samples were obtained from the repository provided by Qin et al. (2012), deposited in the NCBI Sequence Read Archive under accession numbers SRA045646 and SRA050230, and categorized into disease states based on the associated metadata. The raw sequences were subject to quality filtering steps, which were described in the SOP of the Human Microbiome Project Consortium (2012). After preprocessing, using MetaPhlAn2 taxonomic classification tool, metagenome samples were assigned to its microbial species of origin (taxa) and the relative abundance composition of each taxon of a sample was inferred accordingly. These taxa and their relative abundances formed the features to be employed in the machine learning algorithms. As illustrated in Table 1, the data consist of 290 samples and 1,455 microbial species. One hundred thirty-five of the samples are T2D patients, and 155 are healthy. Table 1 presents some lines of the metagenomics dataset for T2D, following the initial preprocessing of the original data. The relative abundance values of each species for each sample are shown in this dataset. The features correspond to different species including bacteria, viruses, and archea. The samples have one of the two class labels, i.e., healthy (shown with 0) and T2D patient (shown with 1).

**TABLE 1:** The metagenomics dataset of T2D, after the initial preprocessing of the original metagenomics data.

	*Methanobrevibacter smithii*	*Methanosphaera*	*Acidobacteriaceae*	*…*	*Megasphaera* sp. BV3C16	Class label (healthy/T2D patient)
Sample 1	0.334	0	0		0	0 (Healthy)
Sample 2	0.141	0	0	0.632	0.03	1 (T2D patient)
…						
Sample 290						

*The relative abundance values of each species for each sample are shown in this dataset. The features correspond to different species including bacteria, viruses, and archea. The samples have one of the two class labels, i.e., healthy (shown with 0) and T2D patient (shown with 1).*

**FIGURE 1:**
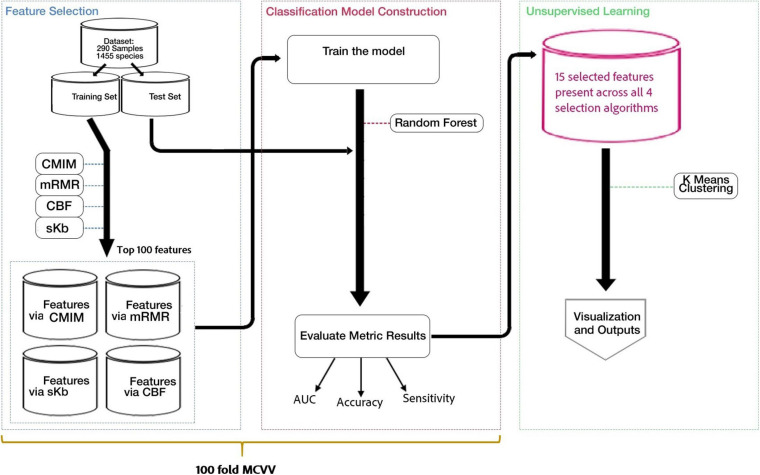
Flowchart of our method, including three main parts. (i) Feature selection methods are applied to detect the most important species for the development of T2D (T2D-associated microorganisms). (ii) Using the selected features, models are constructed and used for classification. (iii) *K*-means clustering algorithm is applied on data to specify subgroups of patients and control samples.

Figure 1 shows the workflow of our methodology. As shown in Figure 1, the following flowchart is applied: (i) the application of feature selection to detect the most important species for the development of T2D (T2D-associated microorganisms), (ii) model construction and classification, and (iii) application of clustering algorithms to specify subgroups of patients and control samples.

### Feature Selection

The dimension of the data is 1,455 (1,455 microbial species) that might influence the performance of the classification algorithms. Thus, a feature selection process is necessary to reduce the dimension of the model and make it also easier for classification and for interpretation. In order to select informative features, in other words to reduce the number of taxa (species), min Redundancy Max Relevance (mRMR) (Brown et al., 2012), Lasso (Tibshirani, 1996), Elastic Net (Zou and Hastie, 2005), and iterative sure select algorithm (Duvallet et al., 2017) have been applied in literature.

We suggest that using some feature selection algorithms such as Peng’s mRMR (Brown et al., 2012), Fleuret’s CMIM (Fleuret, 2004), FCBF (Senliol et al., 2008), and SKB (Pedregosa et al., 2011) could improve classification performance, and by reducing the number of features, we can detect candidate taxonomic biomarkers.

Basically, the mRMR (Brown et al., 2012) method aims to select the features that have the least correlation between themselves (min redundancy) and the highest correlation with a class to predict (max relevance). In order to find the best subset of features, this method starts with an empty set and uses mutual information to weight features and forward selection technique with sequential search strategy. It is a multivariate feature selection method, which calculates the dependency between each feature pair, in addition to class relevance.

Conditional Mutual Information Maximization (Fleuret, 2004) determines the importance of features based on their conditional entropy and mutual information with the class. If the feature carries additional information, it selects that feature. Similarly, FCBF (Senliol et al., 2008) ranks features based on their mutual information with the class to predict, and then removes the features whose mutual information is less than a predefined threshold. It uses the idea of “predominant correlation”. It selects features in a classifier-independent manner, selecting features with high correlation with the target variable, but little correlation with other variables. Notably, the correlation used here is not the classical Pearson or Spearman correlations, but Symmetrical Uncertainty (SU). SU is based on information theory, drawing from the concepts of Shannon entropy and information gain. In other words, FCBF aims at reducing redundancy among selected features. FCBF provides an interpretable and robust option, with results that are generally good. The application of filter-based feature selections for big data analysis in the biomedical sciences not only can have a direct effect in classification efficiency but also might lead to interesting biological interpretations and possible quick identification of biomarkers.

Select K best scores the features against the class label using a function and selecting features according to the *k* highest score (Pedregosa et al., 2011). CMIM, mRMR, FCBF, and SKB feature selection methods are applied using the skfeature and sklearn libraries in Python 3^1^.

Hacilar et al. (2019) applied some of these feature selection methods on inflammatory bowel disease-associated metagenomics dataset and reported to obtain good performance metrics. Most of those feature selection approaches are well studied and well known to achieve good results in human microbiome studies, as reported in a recent review (Marcos-Zambrano et al., 2021).

### Classification Model Construction

In order to evaluate the effects of different classification methods, in our preliminary analysis, we have used Decision Tree, RF, LogitBoost, AdaBoost, an ensemble of SVM with kNN (*k* nearest neighbor), and an ensemble of the Logitboost with kNN. Since the tree model is easy for interpretation and since one can easily convert the model into rule set, in our further experiments, we continued with RF. Additionally, RF is one of the most used algorithms in the human microbiome studies as reported by Marcos-Zambrano et al. (2021).

We designed our actual experiments as follows. We used 100-fold Monte Carlo cross-validation (MCCV), which is the process of randomly selecting (without replacement) some fraction of the data to generate the training set and then assigning the rest to the test set (Xu and Liang, 2001). This process is repeated multiple times, and new training and test partitions are randomly generated each time. We have chosen 90% for training and 10% for testing. As shown in Figure 1, the feature selection methods are applied on the training set.

The Konstanz Information Miner (KNIME) platform (Berthold et al., 2008) is used for the implementation of our methodology. We used the RF predictor node from H20 library in KNIME.

### Model Performance Evaluation

In order to evaluate model efficiency, we measured a range of statistical measures such as sensitivity, specificity, accuracy, and F1 measure for each created model. In this respect, we used the following formulations:

(1) S⁢e⁢n⁢s⁢i⁢t⁢i⁢v⁢i⁢t⁢y⁢(R⁢e⁢c⁢a⁢l⁢l)=T⁢r⁢u⁢e⁢P⁢o⁢s⁢i⁢t⁢i⁢v⁢e⁢/⁢(T⁢r⁢u⁢e⁢P⁢o⁢s⁢i⁢t⁢i⁢v⁢e+F⁢a⁢l⁢s⁢e⁢N⁢e⁢g⁢a⁢t⁢i⁢v⁢e)

(2) P⁢r⁢e⁢c⁢i⁢s⁢i⁢o⁢n=T⁢r⁢u⁢e⁢P⁢o⁢s⁢i⁢t⁢i⁢v⁢e⁢/⁢(T⁢r⁢u⁢e⁢P⁢o⁢s⁢i⁢t⁢i⁢v⁢e+F⁢a⁢l⁢s⁢e⁢P⁢o⁢s⁢i⁢t⁢i⁢v⁢e)

(3) S⁢p⁢e⁢c⁢i⁢f⁢i⁢c⁢i⁢t⁢y=T⁢r⁢u⁢e⁢N⁢e⁢g⁢a⁢t⁢i⁢v⁢e⁢/⁢(T⁢r⁢u⁢e⁢N⁢e⁢g⁢a⁢t⁢i⁢v⁢e+F⁢a⁢l⁢s⁢e⁢P⁢o⁢s⁢i⁢t⁢i⁢v⁢e)

(4) $$F\,1-m\,e\,a\,s\,u\,r\,e=\left(2^{*}\,P\,r\,e\,c\,i\,s\,i\,o\,n^{*}\,R\,e\,c\,a\,l\,l\right)\,/\,\left(P\,r\,e\,c\,i\,s\,i\,o\,n+R\,e\,c\,a\,l\,l\right)$$

(5) Accuracy=(TruePositive+TrueNegative)/(TruePositive+TrueNegative+FalsePositive+FalseNegative).

Additionally, the area under the receiver operating characteristic (ROC) curve (AUC) is used to approximate the probability of the classifier that would score a randomly selected positive instance higher than a randomly selected negative instance.

The average of 100-fold MCCV (Xu and Liang, 2001) results is reported for all performance measures.

### Unsupervised Learning

In order to find subgroups of patients and subgroups of healthy people, we have applied the *k*-means algorithm. *k*-means (Steinley and Brusco, 2007) is an unsupervised clustering algorithm that groups the data into clusters based on similarity or distance metric. *k*-means algorithm minimizes the error inside groups and maximizes the distance between the clusters. We have considered the Euclidean distance metric in our analysis. We used the Elbow method^2^ to determine the optimum number of clusters. In this method, the slow down point denotes the optimum number of clusters.

## Experiments

### Feature Selection and Classification

We have 1,455 features in our data, and we investigated for irrelevant and uninformative features. For this purpose, we applied four most well-studied feature selection algorithms, which are CMIM, mRMR, FCBF, and SKB. In our preliminary analysis, in order to evaluate the effects of different classification methods, Decision Tree, RF, LogitBoost, AdaBoost, an ensemble of SVM with kNN (*k* nearest neighbor), and an ensemble of the Logitboost with kNN are applied. As shown in Supplementary Table 1 and Supplementary Figure 1, RF classifier generated the best performance results and we decided to continue with this classifier in our further experiments.

**FIGURE 2:**
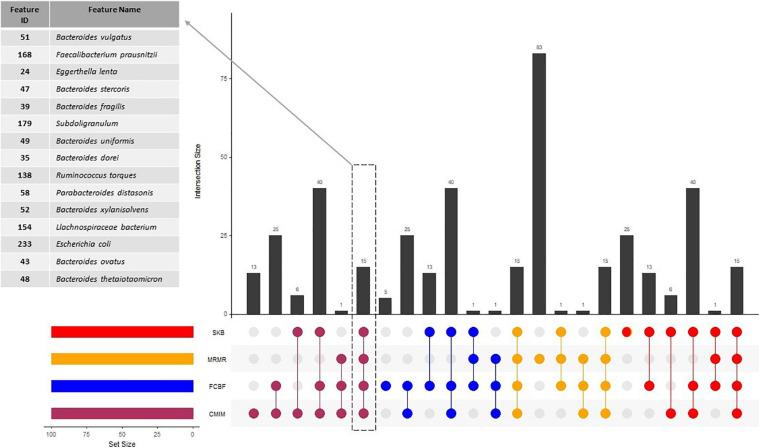
Numbers of features, which are selected by different feature selection algorithms. The commonalities between the selected features by different methods are also illustrated.

At the end of our experiments with 100-fold MCCV and RF classifier (as shown in Figure 1), we have listed the top 100 and top 500 identified features for each feature selection method in Supplementary Tables 2, 3, respectively. The commonalities between those top 100 and top 500 identified feature sets are investigated, and the commonly detected 15 and 199 features within top 100 and top 500 identified features are shown in Supplementary Tables 2, 3, respectively. The commonalities between top 100 identified feature sets, and the details of the 15 features, which are selected by all of the feature selection methods, are shown in Figure 2. In addition to the commonalities in species level, we investigated the commonalities in genus level. Nineteen genera are selected by all of the feature selection methods, as shown in Supplementary Figure 2.

**FIGURE 3:**
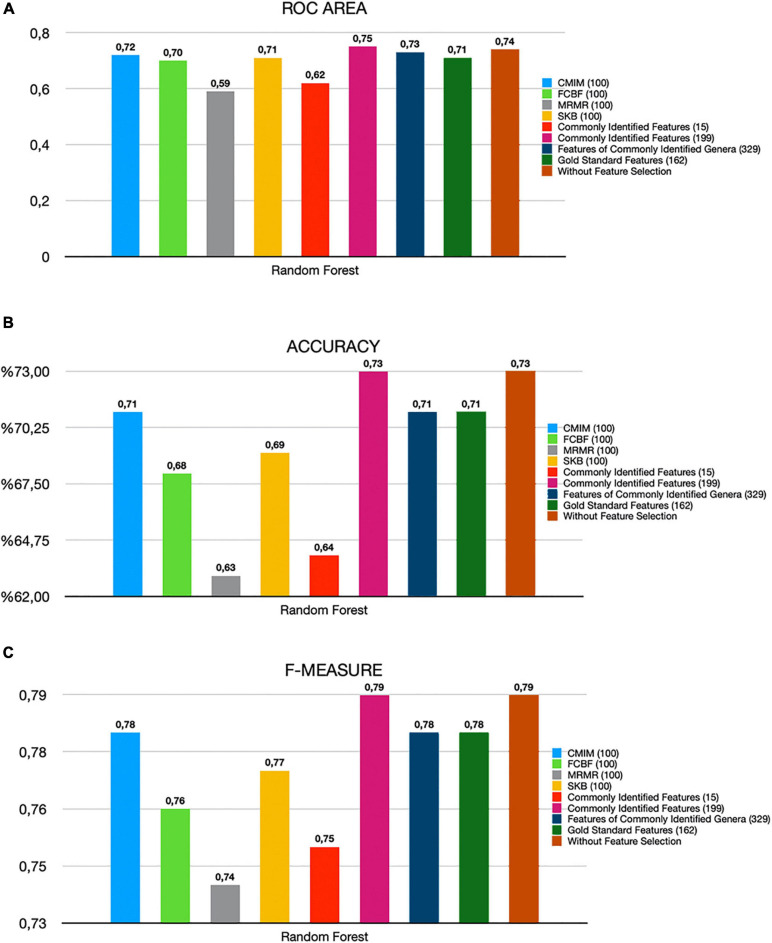
Comparative evaluation of different feature selection methods based on **(A)** ROC area, **(B)** accuracy, and **(C)** F-measure metrics.

By using several metrics as described in section “Model Performance Evaluation”, we have compared the performances of (i) all features (without feature selection); (ii) top 100 and top 500 features selected using CMIM, mRMR, FCBF, and SKB; (iii) 15 and 199 features that are common among top 100 and top 500 features of all four tested feature selection methods; (iv) 329 identified features of 19 commonly detected genera in all four tested feature selection methods (Supplementary Table 4); and (v) 162 features of the gold standard genera that are reported to be associated with T2D in Gurung et al. (2020), as shown in Supplementary Table 5. A detailed comparative evaluation of our findings is presented in Table 2 and Figure 3. As shown in Figure 3, the generated RF model resulted in 0.79 F1-score, 0.74 AUC, and 73% accuracy when all 1,455 features are used (without applying feature selection methods). On the other hand, when 199 features that are commonly selected in the top 500 features of all feature selection methods are used, the generated RF model resulted in 0.79 F1-score, 0.75 AUC, and 73% accuracy. Those selected 199 features performed as good as all features, even 1% higher in terms of AUC metric. Those selected 199 features also performed better compared to the performance (0.78 F1-score, 0.71 AUC, and 71% accuracy) of the 162 features (species) that belong to the gold standard genera, which are reported to be associated with T2D in a recent review paper (Gurung et al., 2020). By only using the 15 features that are commonly selected in the top 100 features list of all four tested feature selection methods, 0.75 F1-score, 0.62 AUC, and 64% accuracy metrics were obtained. In other words, T2D diagnosis could be possible with 64% accuracy by checking only the amounts of 15 specific species among 1,455 different species. As shown in Figure 3, the model using only those 15 species resulted in almost the same F1-score (0.75), with the F1-score obtained using all features (0.79). Checking the amounts of fewer features means less time and cost. In this respect, only using 15 features yielded comparable evaluation metrics.

**TABLE 2:** Comparative evaluation of the different feature selection methods, based on different performance metrics.

Methods		Accuracy	Recall	Specificity	Precision	AUC	F1	Number of features
CMIM	Score	0.71	0.90	0.48	0.72	0.72	0.78	100
	Std. dev.	0.10	0.11	0.34	0.15	0.11	0.05	
	Score	0.73	0.89	0.53	0.72	0.74	0.78	500
	Std. dev.	0.08	0.11	0.25	0.12	0.07	0.04	
FCBF	Score	0.68	0.91	0.41	0.68	0.70	0.76	100
	Std. dev.	0.08	0.10	0.27	0.10	0.09	0.04	
	Score	0.72	0.91	0.48	0.71	0.74	0.78	500
	Std. dev.	0.09	0.10	0.28	0.12	0.09	0.05	
MRMR	Score	0.63	0.95	0.23	0.62	0.59	0.74	100
	Std. dev.	0.06	0.12	0.27	0.10	0.12	0.02	
	Score	0.73	0.86	0.57	0.74	0.74	0.78	500
	Std. dev.	0.07	0.11	0.28	0.14	0.08	0.03	
SKB	Score	0.69	0.91	0.41	0.68	0.71	0.77	100
	Std. dev.	0.08	0.10	0.27	0.10	0.09	0.04	
	Score	0.71	0.92	0.46	0.69	0.74	0.78	500
	Std. dev.	0.08	0.08	0.25	0.10	0.09	0.04	
Commonly identified species (using top 100 features of each feature selection method)	Score	0.64	0.96	0.25	0.62	0.62	0.75	15
	Std. dev.	0.06	0.06	0.19	0.06	0.1	0.03	
Commonly identified species (using top 500 features of each feature selection method)	Score	0.73	0.89	0.54	0.73	0.75	0.79	199
	Std. dev.	0.08	0.09	0.25	0.11	0.09	0.05	
Identified species of commonly detected genus names	Score	0.71	0.91	0.46	0.70	0.73	0.78	329
	Std. dev.	0.09	0.09	0.28	0.11	0.09	0.05	
Species of gold standard genera of T2D	Score	0.71	0.91	0.46	0.70	0.71	0.78	162
	Std. dev.	0.09	0.11	0.28	0.11	0.10	0.05	
All features	Score	0.73	0.89	0.52	0.72	0.74	0.79	1,455
	Std. dev.	0.08	0.09	0.26	0.11	0.09	0.05	

### Feature Correlations

**FIGURE 4:**
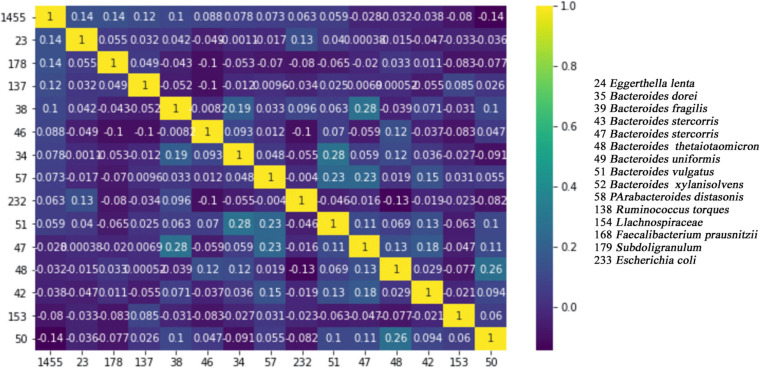
Pairwise correlation heat map of 15 commonly identified features. While number 1 (shown in yellow) indicates full correlation, number 0 (shown in dark blue) indicates no correlation.

The pairwise correlations of 15 features, which are commonly selected by all four tested feature selection methods, may be important for the further studies of T2D in terms of developing probiotics. For this reason, we have calculated the pairwise correlations of those 15 selected features using the tool in^3^, and we have generated a heat map, as presented in Figure 4. It can be concluded from Figure 4 that there are no important positive correlations between any two species among any two pairs of 15 selected species. This result indicates that each one of the selected 15 features has its own information and each feature (species) has an independent contribution to T2D development.

### Clustering

**FIGURE 5:**
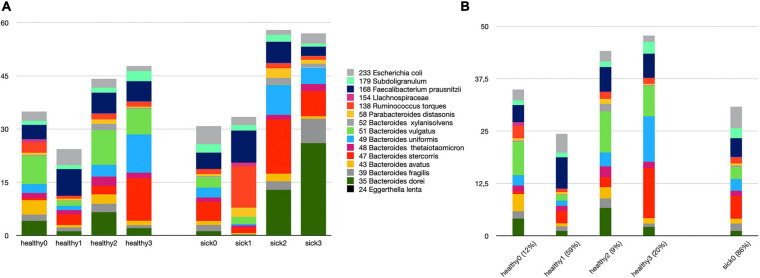
The relative amounts of 15 species **(A)** in all healthy and T2D subgroups. **(B)** Zoomed-in view of all healthy subgroups and one T2D subgroup, which covers more than 86% of all samples.

**FIGURE 6:**
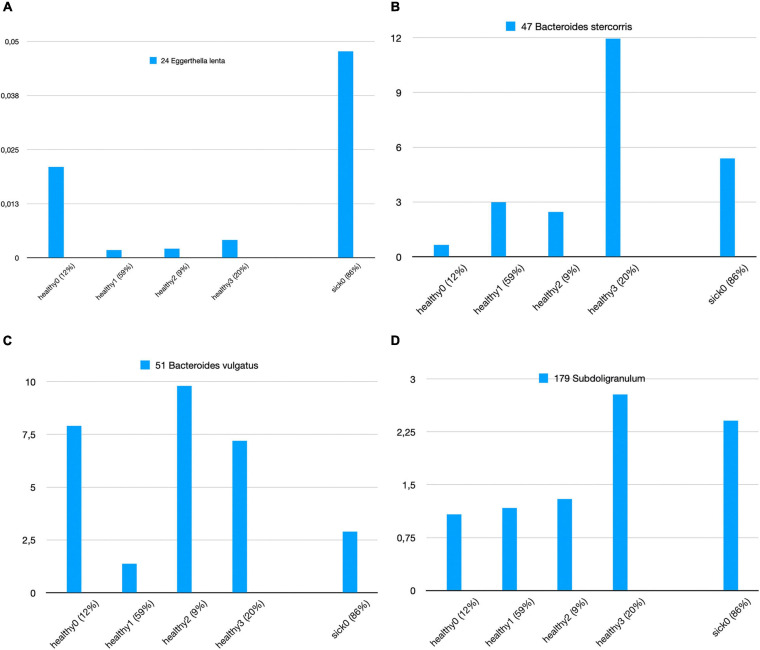
Zoomed-in view of all healthy subgroups and the biggest T2D subgroup for **(A)** *Eggerthella lenta*, **(B)** *Bacteroides stercoris*, **(C)** *Bacteroides vulgatus*, and **(D)** *Subdoligranulum*.

We attempt to answer whether there could be any direct relationship between specific species and T2D subgroups. In order to answer this question, we used *k*-means clustering algorithm and subgrouped the healthy samples and sick samples separately. As shown in Supplementary Figure 3, we decided to generate four subgroups for healthy samples and four subgroups for sick samples. Figure 5 illustrates the identified healthy and T2D subgroups and the presence of the species in each of these subgroups. In Figure 6, we displayed more in detail the presence of four selected species in each of the healthy subgroups and one T2D subgroup, which covers 86% of the T2D patients from our dataset. It can be concluded from Figures 5, 6 that even though the samples were divided into subgroups, a single species may not have a direct effect on the development of T2D for a specific group. Nevertheless, there are a few observations that we can make: (i) *Bacteroides vulgatus* (shown in green in Figures 5A, 6C) is mainly observed in healthy subgroups (healthy 0, 2, and 3) and found in reduced amounts in T2D patients. (ii) *Eggerthella lenta* is observed in reduced amounts in all healthy subgroups compared to the biggest subgroup of T2D patients (sick0), which includes 86% of the T2D patients from our dataset (shown in Figure 6A). (iii) *Bacteroides stercoris* (shown in red in Figure 5A) is present in reduced amounts in three of the healthy groups (healthy 0, 1, 2), compared to the biggest subgroup of T2D patients (sick0 in Figure 6B). (iv) Similarly, *Subdoligranulum* (shown in light green in Figure 5B) is present in reduced amounts in three of the healthy groups (healthy 0, 1, and 2), compared to the biggest subgroup of T2D patients (sick0 in Figure 6D).

## Discussion

The human gut microbiome contains trillions of living species. T2D is a disease that affects approximately 500 million people in the world. Like many other diseases, T2D might have a special association with gut microbiota (Manor et al., 2020). In the last decade, the identification of gut microbiota related to T2D has served as a stimulus for exponential advances in scientific production (Gurung et al., 2020). Multiple factors are reported to be involved in the changes of gut microbiota and hence its relationship to T2D (Sharma and Tripathi, 2019). The contribution of various molecular mechanisms of gut microbiota to T2D has been recently reviewed in Aw and Fukuda (2018). In order to change the gut microbiota to our benefit, several possibilities are currently available, and these possibilities are providing encouraging results. In this respect, in this study, by analyzing the T2D-associated metagenomics data using several supervised and unsupervised machine learning algorithms, we attempt to discover potential taxonomic biomarkers of T2D. Our metagenomics dataset includes the amounts of 1,455 species, measured on the gut microbiota of 290 humans. We used different feature selection algorithms including CMIM, mRMR, FCBF, and SelectKBest. In our preliminary study, we used different classification algorithms including RF, Decision Tree, LogitBoost, AdaBoost, SVM + *k* means, and Logitboost + *k* means. In these preliminary experiments, as shown in Supplementary Table 1 and Supplementary Figure 1, we observed that RF resulted in best performance metrics and we decided to continue with our experiments using RF classifier.

All tested feature selection methods commonly identified 15 specific features (as shown in Figure 2). Using the amounts of these 15 features, our generated model with RF could predict the T2D status of a sample with 64% accuracy. Compared to the 73% accuracy level using all 1,455 features, 73% accuracy level using 199 selected features, and 71% accuracy level using 162 gold standard features, these 15 selected features yielded reasonable accuracy results with much lower features. Also, the model using only those 15 species resulted in almost the same F1-score (0.75), with the F1-score obtained using all features (0, 79), as shown in Figure 3. Hence, these features could be further evaluated as potential taxonomic biomarkers of T2D. The identified features are *Bacteroides dorei*, *Bacteroides fragilis*, *Bacteroides ovatus*, *Bacteroides stercoris*, *Bacteroides thetaiotaomicron*, *Bacteroides uniformis*, *Bacteroides vulgatus*, *Bacteroides xylanisolvens*, *E. lenta*, *Escherichia coli*, *Faecalibacterium prausnitzii*, *Lachnospiraceae bacterium*, *Parabacteroides distasonis*, *Ruminococcus torques*, and *Subdoligranulum*. The associations of most of these features with T2D is also reported in literature as follows.

A recent review paper (Gurung et al., 2020) summarized the potential mechanisms of microbiota and its effects on the metabolism of T2D patients. Briefly, microbiota modulates inflammation, interacts with dietary constituents, and affects gut permeability, glucose and lipid metabolism, insulin sensitivity, and overall energy homeostasis in the mammalian host. In that study, Gurung et al. highlighted specific taxa that can affect T2D and presented the possible roles of these species in terms of T2D development. They surveyed 42 human observational studies on T2D and the bacterial microbiome, and they reported Bacteroides as the second most commonly reported genus (Gurung et al., 2020). The studies that investigated this genus on the species level indicated that the levels of *Bacteroides intestinalis*, *Bacteroides 20-3*, and *Bacteroides vulgatus* were dropped in T2D patients, and the levels of *Bacteroides stercoris* were increased after sleeve gastrectomy surgery in T2D patients with diabetes remission (Wu, 2010; Karlsson et al., 2013; Zhang et al., 2013; Murphy et al., 2017). Additionally, two experimental animal studies tested the ability of *Bacteroides* in order to treat diet-induced metabolic disease (Cano, 2012; Yang, 2017). These studies indicated that the administration of *Bacteroides acidifaciens* (Yang, 2017) and *Bacteroides uniformis* (Cano, 2012) improved glucose intolerance and insulin resistance in diabetic mice. In another study, using a mouse model, Yoshida et al. (2018) found that *B. vulgatus* and *B. dorei* upregulates the expression of tight junction genes in the colon, which leads to reduction in gut permeability, reduction of lipopolysaccharides production, and amelioration of endotoxemia. T2D is known to be associated with increased levels of pro-inflammatory cytokines, chemokines, and inflammatory proteins (Gurung et al., 2020). Along this line, using mono-associated mice, Hoffman et al. (2016) reported that *Bacteroides thetaiotaomicron* reduces Th1, Th2, and Th17 cytokines. Chang et al. (2017) demonstrated that the induction of IL-10 by *Bacteroides fragilis* may contribute to the improvement of glucose metabolism because the overexpression of this cytokine in muscle protects from aging-related insulin resistance (Dagdeviren, 2017; Gurung et al., 2020). Taken together, these studies indicate that *Bacteroides* species play a beneficial role on glucose metabolism in humans and experimental animals. Among these *Bacteroides* species, *B. dorei*, *B. fragilis*, *B. stercoris*, *B. thetaiotaomicron*, *B. uniformis*, and *B. vulgatus* are identified among the top 15 features list in our study. In addition to these species as potential taxonomic biomarkers of T2D, in this study, we suggest *B. ovatus* and *B. xylanisolvens* as two potential taxonomic biomarkers of T2D. Among the abovementioned *Bacteroides* species, *B. intestinalis*, *B. 20-3*, and *B. acidifaciens* did not exist in our top 15 species list.

In addition to the genera of *Bacteroides*, the effect of *Faecalibacterium* genus with respect to T2D development is discussed in the same review paper by Gurung et al. (2020). Gao et al. (2018) and Salamon et al. (2018) reported the lower frequencies of *Faecalibacterium* in the disease group of case–control study on T2D. While this genus was mostly reported to be decreased after different types of antidiabetic treatments ranging from metformin and herbal medicine (Tong et al., 2018) to bariatric surgery (Murphy et al., 2017), one study reported an opposite effect (Patrone et al., 2016). The studies that investigate this genus at species level usually detected *Faecalibacterium prausnitzii. F. prausnitzii* and the peptides secreted by this bacterium are shown to perform anti-inflammatory effects in different chemically induced colitis models in mice (Sokol et al., 2008; Quévrain et al., 2016; Breyner et al., 2017). In different human case–control studies, *F. prausnitzii* was found to be negatively associated with T2D (Furet et al., 2010; Graessler et al., 2013; Karlsson et al., 2013; Zhang et al., 2013; Remely et al., 2014). Although *F. prausnitzii* is commonly used as a probiotic for colitis (Rossi et al., 2015), only a few studies suggested using *F. prausnitzii* as a probiotic for metabolic disease. As shown in Figure 2, our top 15 features list includes *F. prausnitzii* and we suggest it as a potential taxonomic biomarker of T2D.

The genera of *Ruminococcus* has also been reported to have a positive association with T2D in the recent review paper by Gurung et al. (2020). Gurung et al. added that the studies reporting species levels of these bacteria reported conflicting results (Graessler et al., 2013; Murphy et al., 2017; Wu et al., 2017). For example, while Wu et al. (2017) found that *Ruminococcus* sp. *SR1/5* is enriched by metformin treatment, Murphy et al. (2017) demonstrated that *Ruminococcus bromii* is enriched and *Ruminococcus torques* is decreased after bariatric surgery and diabetes remission. Among these *Ruminococcus* species, *Ruminococcus torques* is identified among the top 15 features list in our study.

A recent study by Wang et al. (2019) demonstrated that *P. distasonis* prevents obesity and metabolic dysfunctions by producing succinate and secondary bile acids. Using ob/ob and high-fat diet-fed mice, they showed the metabolic benefits of *P. distasonis* in terms of decreasing weight gain, hyperglycemia, and hepatic steatosis. As shown in Figure 2, we detected *P. distasonis* in the top 15 features list in our study and we suggested it as a potential taxonomic biomarker of T2D.

Recently, the metformin treatment, which is the most prescribed antidiabetic drug, is shown to disturb the intestinal microbes. Hence, the compositional shifts were detected in the representative gut microbiomes of T2D patients undergoing metformin treatment. *Subdoligranulum variabile* is one of those microbes that is found to display increased abundance in those T2D patients undergoing metformin treatment (Forslund et al., 2015; Mardinoglu et al., 2016; Wu et al., 2017). As shown in Figure 2, we identified *S. variabile* in the top 15 features list.

Qin et al. (2012) demonstrated that the opportunistic pathogens (e.g., *Clostridium hatheway*, *Bacteroides caccae*, *E. coli*, and *E. lenta*) are increased in diabetes. On the other hand, Doumatey et al. (2020) reported that they did not find any evidence of such enrichment in their study, where they analyzed the gut microbiome profiles of T2D patients in Urban Africans. As shown in Figure 2, our top 15 features list includes *E. coli* and *E. lenta*. Although our top 15 features list did not include *C. hatheway*, different strains of this species are identified by all four tested feature selection methods, as shown in Supplementary Tables 2, 4. We realized that different strains of this species such as *C. hathewayi_GCF_000160095*, *Clostridium hathewayi_GCF_000235505*, and *C. hathewayi unclassified* are detected in the top 100 lists of all four tested feature selection methods, as shown in Supplementary Table 2. Also, increased levels of *C. clostridioforme* in T2D patients are reported by Karlsson et al. (2013) and Qin et al. (2012). In our study, *C. clostridioforme* is included within the 199 commonly identified features of top 500 selected features, as shown in Supplementary Table 3, and the genera of *Clostridium* is identified by all tested feature selection methods, as shown in Supplementary Figure 2.

*Lachnospiraceae* species constitute the core of gut microbiota. They colonize the intestinal lumen from the birth, and during the host’s life, they increase both in terms of the diversity of their species and their relative abundances. Although they are commonly found in the gut microbiota and their members are among the main producers of short-chain fatty acids, different *Lachnospiraceae* species are also associated with different intra- and extraintestinal diseases (Vacca et al., 2020). Kostic et al. (2015) reported that *Lachnospiraceae* genus negatively affects glucose metabolism, which leads to inflammation and promotes the onset of T1D. Along this line, using both human and mouse models, some other metagenomics studies demonstrated that *Lachnospiraceae* may also be specifically associated with T2D (Qin et al., 2012; Kameyama and Itoh, 2014). As shown in Figure 2, we detected *Lachnospiraceae* in the top 15 features list in our study.

The recent review paper by Gurung et al. (2020) pointed out that in addition to the genera of *Bacteroides*, the genera of *Bifidobacterium* is another beneficial genera and it is most frequently reported in the studies of T2D. They reported that the genera of *Bifidobacterium* is most consistently supported by the literature in terms of containing the microbes potentially protective against T2D (Gurung et al., 2020). For example, Wu et al. (2017) and Murphy et al. (2017) found a negative association between *Bifidobacterium adolescentis*, *Bifidobacterium bifidum*, *Bifidobacterium pseudocatenulatum*, *Bifidobacterium longum*, *Bifidobacterium dentium*, and disease in patients treated with metformin or after undergoing gastric bypass surgery. Although *Bifidobacterium* has not been used alone as probiotics for T2D, most of the animal studies that tested different species from this genus (*B. bifidum*, *B. longum*, *B. infantis*, *B. animalis*, *B. pseudocatenulatum*, and *B. breve*) showed improvement of glucose tolerance (Le, 2015; Moya-Perez et al., 2015; Wang, 2015; Aoki, 2017; Kikuchi et al., 2018). These studies strengthen the idea that *Bifidobacterium* naturally habituating the human gut or introduced as probiotics play a protective role in T2D. In our study, several *Bifidobacterium* species (including *B. bifidum*, *B. longum*, *B. pseudocatenulatum*, *B. breve*, *B. animalis*, *B. adolescentis*, and *B. dentium*) are found as important features in the top 100 features lists of each one of four tested feature selection methods (as can be seen in Supplementary Table 2). However, each feature selection method selected a different *Bifidobacterium* species. When we get the intersection of the selected features from four different methods, these *Bifidobacterium* species did not show up in the top 15 selected features list. But on the genus level, *Bifidobacterium* is identified by all feature selection methods (as can be seen in Supplementary Table 2 and Supplementary Figure 2). Once we focus on commonly detected genera instead of commonly detected species in all four tested feature selection methods, these *Bifidobacterium* species showed up among those 329 features, and using these features, 0.78 F1-score, 0.73 AUC, and 71% accuracy performance metrics are obtained, as shown in Figure 3. On the other hand, when we generate the list of top 500 selected features from each feature selection method and check for the commonly identified features among these four lists (as shown in Supplementary Table 3), we end up with 199 commonly selected features. *Bifidobacterium longum*, *B. pseudocatenulatum*, and *B. breve* existed in this list. Classification using these 199 commonly selected features resulted in 73% accuracy, 0.75 ROC, and 0.79 F1-measure, as shown in Figure 3. Those selected 199 features also performed better compared to the performance (0.78 F1-score, 0.71 AUC, and 71% accuracy) of the 162 features (species) that belong to the gold standard genera, which are reported to be associated with T2D in a recent review paper (Gurung et al., 2020). Figure 3 illustrates the comparative evaluation of all the feature selection methods.

Similarly, in our analyses, several *Ruminococcus* species (including *R. gnavus*, *R. obeum*, *R. torques*, *R. albus*, *R. callidus*, *R. sp*, *R. lactaris*, *R. champanellensis*, and *R. flavefaciens*) and several *Blautia* species including *B. hansenii*, *B. producta*, and *B. sp_KLE_1732* are detected as important features in the top 100 features lists of each one of four tested feature selection methods (as can be seen in Supplementary Table 2). Accordingly, these species are included in the identified features list of commonly detected genera in all four tested feature selection methods, shown in Supplementary Table 4. In Gurung et al. (2020), *Ruminococcus*, *Blautia*, and *Fusobacterium* were reported to be positively associated with T2D. The genera of *Fusobacterium* is identified only by SKB feature selection method, as shown in Supplementary Table 4.

On the other hand, two genera (*Akkermansia* and *Roseburia*) that were found to be negatively associated with T2D in Gurung et al. (2020) did not show up in the commonly identified genera list (Supplementary Figure 2). However, these two genera were detected in the top 100 lists of different feature selection methods, as shown in Supplementary Tables 2, 4. As shown in Supplementary Table 4, while the genera of *Akkermansia* is identified by FCBF and SKB feature selection methods, the genera of *Roseburia* is identified by all tested feature selection methods except mRmR.

Pasolli et al. (2016) attempted to classify the T2D patients and healthy samples using the metagenomic-associated dataset of T2D, downloaded from Qin et al. (2012). They followed the same preprocessing as we performed. Before applying MetaPhlAn2, the samples were subject to standard pre-processing as described in the SOP of the Human Microbiome Project. Similar to our study, they used species abundance as input data and tested the performances of the SVM and RF classifiers and also evaluated Lasso and elastic net regularized multiple logistic regression. On T2D-associated metagenomics dataset, without applying any feature selection, they obtained 0.75 F1-score, 0.62 AUC, and 64% accuracy using RF classifier, as shown in Figure 2 of their study. Our RF model without applying feature selection methods resulted in 0.79 F1-score, 0.74 AUC, and 73% accuracy, as shown in Figure 3 and Table 2.

Pasolli et al. (2016) also investigated the effect of different feature selection algorithms. On the T2D-associated metagenomics dataset, by only using 40 species (features) that are selected using Lasso feature selection, Pasolli et al. (2016) obtained 0.70 AUC using RF classifier, as shown in Supplementary Figures 2, 3. In our study, by only using 15 species, 0.74 AUC is obtained using RF classifier, as shown in Figure 3 and Table 2. We can conclude that there is added value in studying T2D through metagenomics and machine learning.

Lastly, we clustered the healthy samples and cases according to these 15 features (the amounts of 15 selected species) using *k*-means clustering. Hence, we attempt to distinguish the subgroups of healthy samples and sick samples. While the relative amounts of 15 selected species are shown in Figure 5 for all healthy and T2D subgroups, in Figure 6, the relative amounts of some specific species are shown for all four healthy subgroups vs. sick0 subgroup, which covers 86% of all the patient samples. Once we evaluated Figures 5, 6, we had some important observations. For example, it can be deduced from Figure 6A that the amount of *E. lenta* in healthy samples is at least 10–11 times less than its amount in patients. Therefore, the abundance of *E. lenta* can be evaluated as a candidate taxonomic biomarker for T2D disorder. Qin et al. (2012) also demonstrated that the levels of opportunistic pathogens such as *E. lenta* are increased in diabetes. Figures 6B–D indicate that *Bacteroides stercoris* (which is numbered as 47), *Bacteroides vulgatus* (which is numbered as 51), and *Subdoligranulum* (which is numbered as 179) can be considered as candidate taxonomic biomarkers of T2D. In literature, the levels of *Bacteroides vulgatus* were reported to be dropped in T2D patients and the levels of *Bacteroides stercoris* were reported to be increased after sleeve gastrectomy surgery in T2D patients with diabetes remission (Wu, 2010; Karlsson et al., 2013; Zhang et al., 2013; Murphy et al., 2017). In another study, using a mouse model, Yoshida et al. found that *B. vulgatus* upregulates the expression of tight junction genes in the colon, which leads to reduction in gut permeability, reduction of lipopolysaccharides production, and amelioration of endotoxemia (57). *Subdoligranulum variabile* is one of those microbes that is found to display increased abundance in those T2D patients undergoing metformin treatment (Forslund et al., 2015; Mardinoglu et al., 2016; Wu et al., 2017).

## Conclusion

Human gut microbiota, which consists of nearly 200 prevalent bacterial species and approximately 1,000 uncommon species, is considered as a multicellular organ. Gut microbiota can affect the host immune system, which is central to program several host activities (Qin et al., 2010). Hence, the metagenomic analysis of the human gut microbiome provides novel insights for several diseases, including T2D. Although several studies reported the significance of the gut microbiota in pathophysiology of T2D, this field is still in its infancy. The existing studies concluded that some microbial taxa and related molecular mechanisms may be involved in glucose metabolism related to T2D. Nevertheless, such simple interpretations are not enough to explain the heterogeneity and complexity of T2D, and the redundancy of gut microbiota further complicates these analyses. Along this line, in this study, we used the T2D-associated metagenomics data and developed a machine learning model to increase the diagnostic accuracy of T2D. We discovered potential taxonomic biomarkers of T2D and investigated which subset of microbiota is more informative than other taxa applying some of the state-of-the art feature selection methods. In our experiments, especially 15 species came into prominence. We present support from literature regarding the association of these species with T2D. Hence, we propose these species as candidate taxonomic biomarkers of T2D, where wet lab scientists can design validation experiments.

## Data Availability Statement

Publicly available datasets were analyzed in this study. This data can be found here: The data is taken from the following paper: Qin et al. (2012). Raw sequencing data is obtained from NCBI Sequence Read Archive with SRA045646 accession number.

## Author Contributions

BB-G conceived the ideas and designed the study. AJ, ON, and MY conducted the experiments. BB-G, OB, AJ, and MY analyzed the results. BB-G, OB, AJ, ON, and MY participated in the discussion of the results and writing of the article. All authors read and approved the final version of the manuscript.

## Conflict of Interest

The authors declare that the research was conducted in the absence of any commercial or financial relationships that could be construed as a potential conflict of interest.

## Publisher’s Note

All claims expressed in this article are solely those of the authors and do not necessarily represent those of their affiliated organizations, or those of the publisher, the editors and the reviewers. Any product that may be evaluated in this article, or claim that may be made by its manufacturer, is not guaranteed or endorsed by the publisher.

## Footnotes

1
https://www.python.org/about/

2
https://predictivehacks.com/k-means-elbow-method-code-for-python/

3
https://github.com/bhattbhavesh91/GA_Sessions/blob/master/ga_dsmp_5jan2019/16_feature_selection.ipynb

## Supplementary Material

Supplementary Figure 1 Preliminary performance evaluation results for T2D-associated metagenomics dataset using 10 fold cross-validation. Comparative evaluation of different classifiers using different feature selection methods based on **(A)** ROC area, **(B)** accuracy, and **(C)** F-measure.

Supplementary Figure 2 Numbers of identified genus, which are selected by different feature selection algorithms. The commonalities between the selected genus in different methods are also illustrated.

Supplementary Figure 3 Selection of the optimum number of the clusters for the **(A)** controls and **(B)** T2D patients. Using the elbow method, four clusters are found as the optimum number of clusters for both the controls [as shown in panel **(A)**] and T2D patients [as shown in panel **(B)**].

Supplementary Table 1 Preliminary analysis results for T2D-associated metagenomics dataset using 10 fold cross-validation. Evaluation of different classification methods based on different performance measures (a) without feature selection and after applying (b) CMIM, (c) mRMR, (d) FCBF, and (e) SKB feature selection algorithms.

Supplementary Table 2 Top 100 selected features for each feature selection method and 15 commonly identified features among these four lists.

Supplementary Table 3 Top 500 selected features for each feature selection method and 199 commonly identified features among these four lists.

Supplementary Table 4 Identified features of commonly detected genera in top 100 lists of all four tested feature selection methods.

Supplementary Table 5 Features of gold standard genera that are reported to be associated with T2D in Gurung et al. (2020).
