# Supplementary figures and images for: Discovering Potential Taxonomic Biomarkers of Type 2 Diabetes From Human Gut Microbiota via Different Feature Selection Methods
Source: Front Microbiol. 2021 Aug 25;12:628426. doi: 10.3389/fmicb.2021.628426 (PMC8424122; doi:10.3389/fmicb.2021.628426)

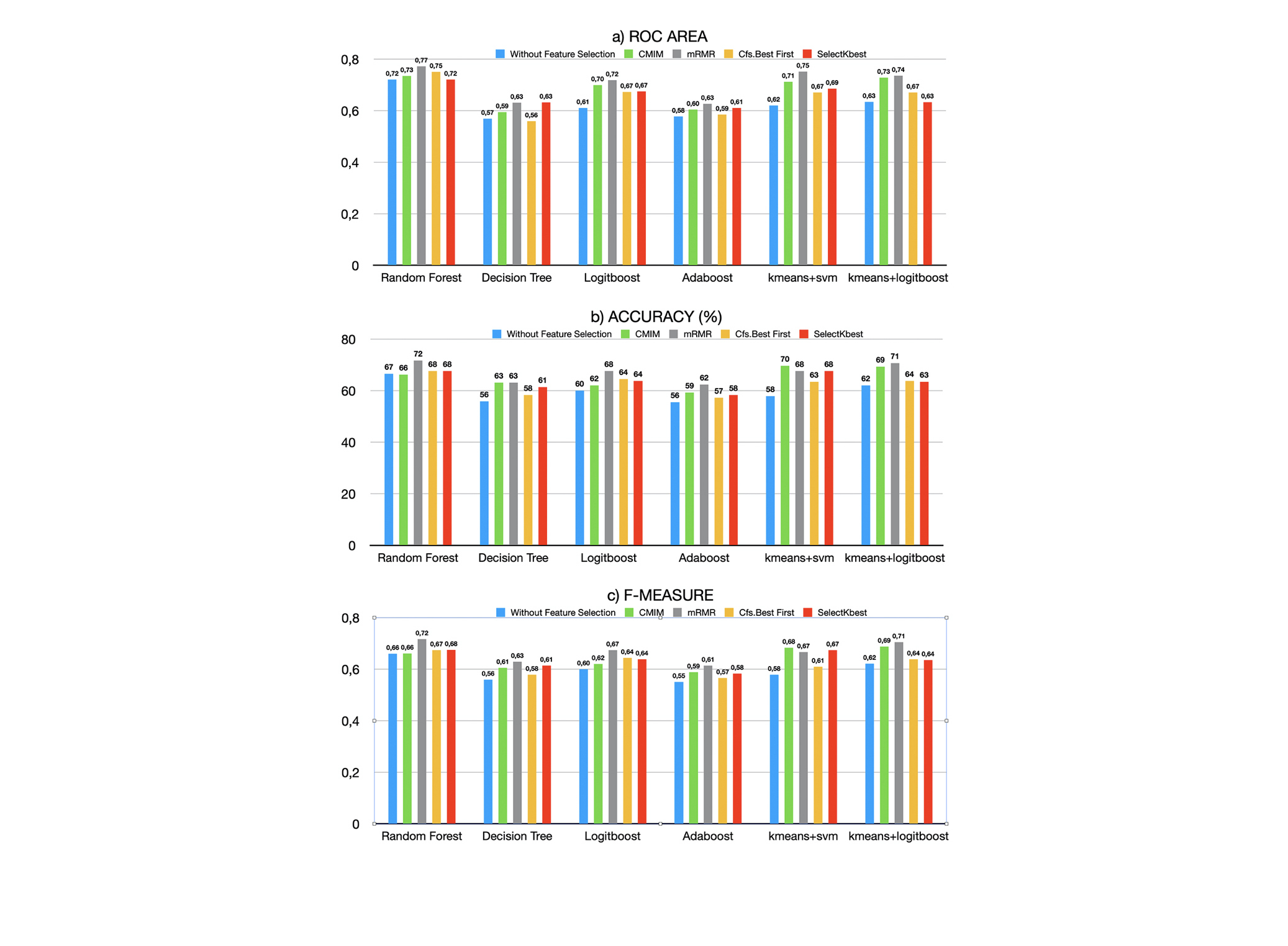

Supplement: Supplementary Figure 1 — Preliminary performance evaluation results for T2D-associated metagenomics dataset using 10 fold cross-validation. Comparative evaluation of different classifiers using different feature selection methods based on (A) ROC area, (B) accuracy, and (C) F-measure. [file Image_1.JPEG]

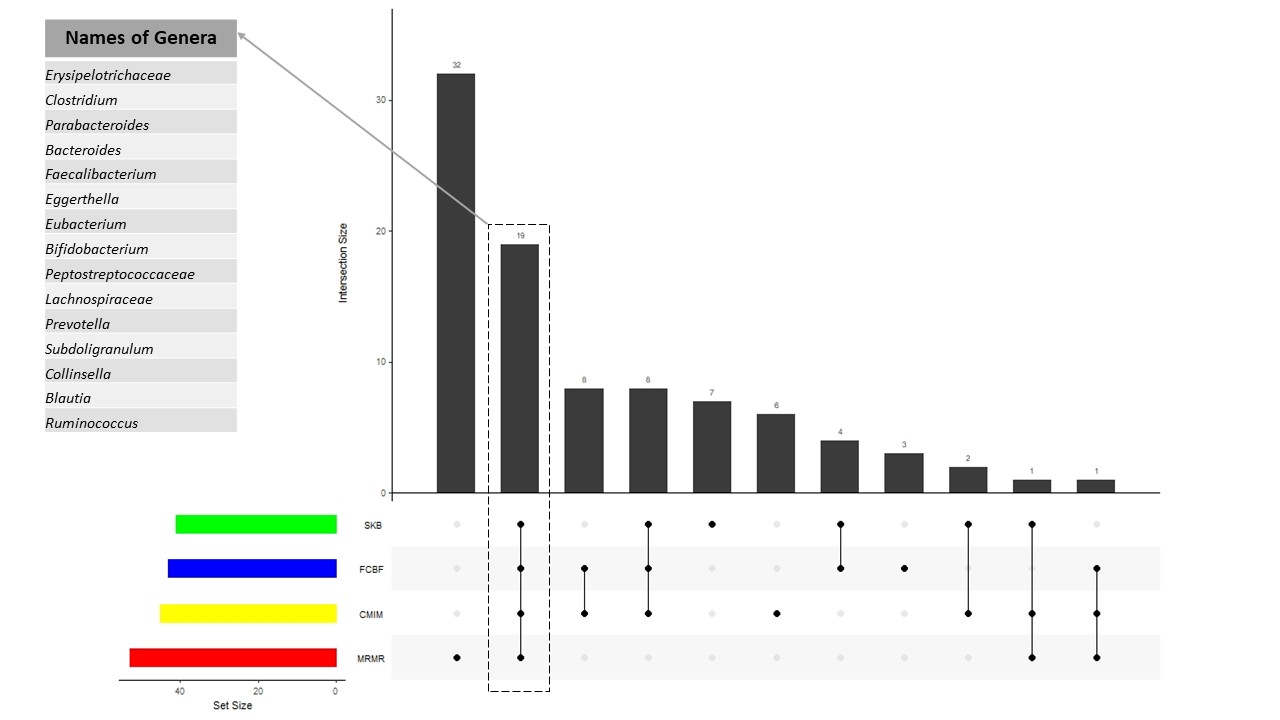

Supplement: Supplementary Figure 2 — Numbers of identified genus, which are selected by different feature selection algorithms. The commonalities between the selected genus in different methods are also illustrated. [file Image_2.JPEG]

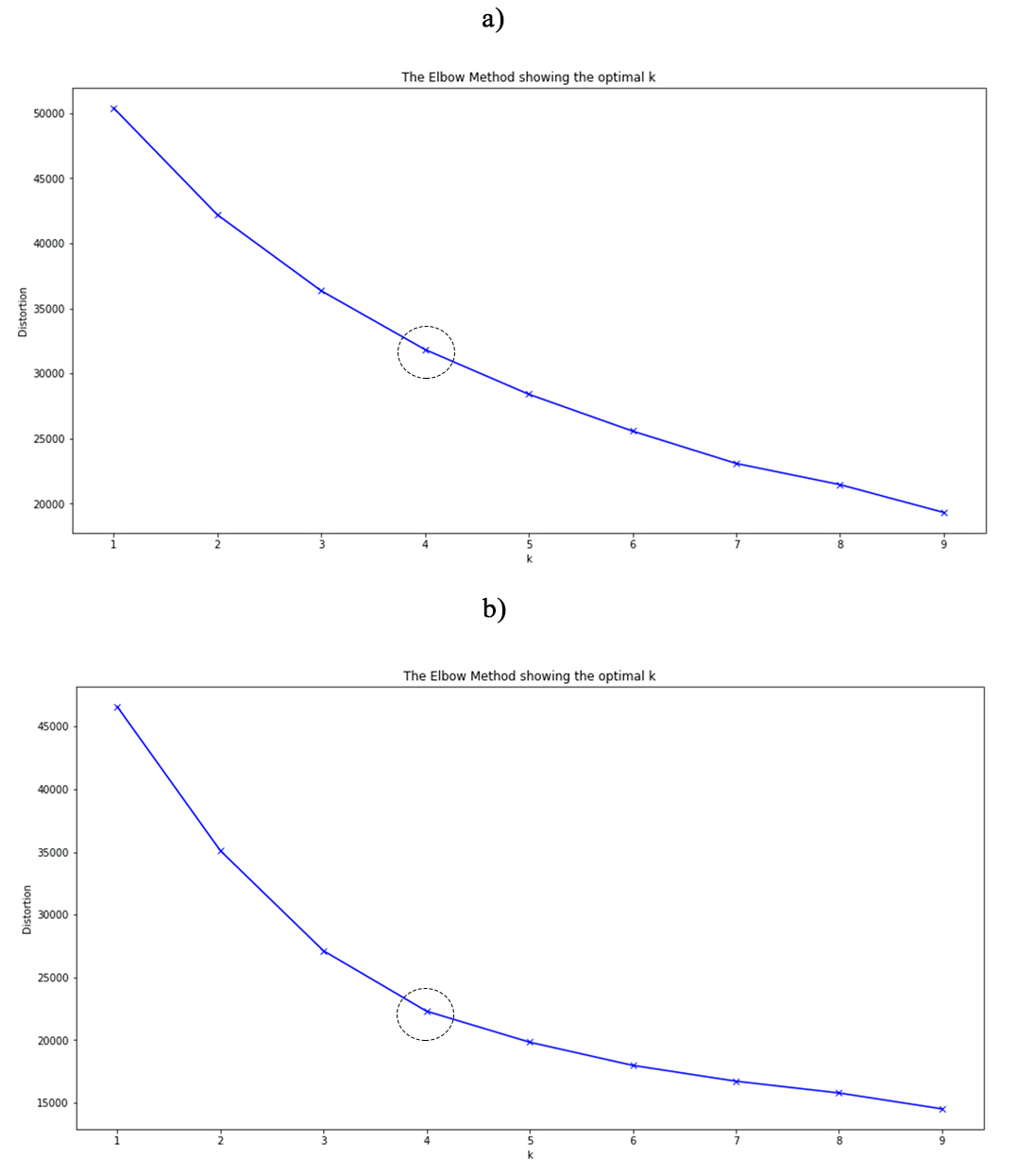

Supplement: Supplementary Figure 3 — Selection of the optimum number of the clusters for the (A) controls and (B) T2D patients. Using the elbow method, four clusters are found as the optimum number of clusters for both the controls [as shown in panel (A)] and T2D patients [as shown in panel (B)]. [file Image_3.JPEG]
