# Supplementary material for: Discovering Potential Taxonomic Biomarkers of Type 2 Diabetes From Human Gut Microbiota via Different Feature Selection Methods
Source: Front Microbiol. 2021 Aug 25;12:628426. doi: 10.3389/fmicb.2021.628426 (PMC8424122; doi:10.3389/fmicb.2021.628426)
Supplement: Supplementary Table 5 — Features of gold standard genera that are reported to be associated with T2D in Gurung et al. (2020). [file Data_Sheet_1.PDF]

a) Without Feature Selection

Classification Algorithm	TP Rate	FP Rate	Precision	Recall	F-Score	ROC	Accuracy
Random Forest	0,666	0,352	0,668	0,666	0,660	0,720	66.5517 %
Decision Tree(J 48)	0,559	0,440	0,562	0,559	0,559	0,569	55.8621 %
LogitBoost	0,600	0,406	0,599	0,600	0,600	0,610	%60
AdaBoost	0,555	0,459	0,552	0,555	0,551	0,577	55.5172 %
kmeans+SVM	0,579	0,429	0,578	0,579	0,578	0,620	%57.931
kmeans+ LogitBoost	0,621	0,378	0,623	0,621	0,621	0,621	%62.069

b) CMIM

Classification Algorithm	TP Rate	FP Rate	Precision	Recall	F-Score	ROC	Accuracy
Random Forest	0,662	0,346	0,661	0,662	0,661	0,734	66.2069 %
Decision Tree(J 48)	0,631	0,404	0,651	0,631	0,605	0,594	63.1034 %
LogitBoost	0,621	0,384	0,620	0,621	0,620	0,699	%62.069
AdaBoost	0,593	0,422	0,591	0,593	0,588	0,604	59.3103 %
kmeans+SVM	0,697	0,330	0,716	0,697	0,683	0,712	69.6552 %
kmeans+ LogitBoost	0,693	0,325	0,698	0,693	0,687	0,728	69.3103 %

c) mRMR

Classification Algorithm	TP Rate	FP Rate	Precision	Recall	F-Score	ROC	Accuracy
Random Forest	0,717	0,289	0,717	0,717	**0,716**	**0,772**	**71.7241 %**
Decision Tree(J 48)	0,631	0,379	0,630	0,631	0,629	0,631	63.1034 %
LogitBoost	0,676	0,337	0,676	0,676	0,673	0,718	67.5862 %
AdaBoost	0,624	0,397	0,626	0,624	0,614	0,626	62.4138 %
kmeans+SVM	0,676	0,346	0,684	0,676	0,666	0,751	67.5862 %
kmeans+ LogitBoost	0,707	0,303	0,707	0,707	0,705	0,735	70.6897 %

d) Cfs.BestFirst (cbf)

Classification Algorithm	TP Rate	FP Rate	Precision	Recall	F-Score	ROC	Accuracy
Random Forest	0,676	0,334	0,675	0,676	0,674	0,750	67.5862 %
Decision Tree(J 48)	0,583	0,431	0,580	0,583	0,578	0,559	58.2759 %
LogitBoost	0,645	0,363	0,644	0,645	0,644	0,672	64.4828 %
AdaBoost	0,572	0,445	0,569	0,572	0,565	0,585	57.2414 %
kmeans+SVM	0,634	0,400	0,655	0,634	0,609	0,670	63.4483 %
kmeans+ LogitBoost	0,638	0,366	0,638	0,638	0,638	0,670	63.7931 %

e) SelectKbest(with ChiSquare)

Classification Algorithm	TP Rate	FP Rate	Precision	Recall	F-Score	ROC	Accuracy
Random Forest	0,676	0,330	0,675	0,676	0,675	0,720	67.5862 %
Decision Tree(J 48)	0,614	0,386	0,616	0,614	0,614	0,632	61.3793 %
LogitBoost	0,638	0,367	0,637	0,638	0,638	0,674	63.7931 %
AdaBoost	0,583	0,417	0,585	0,583	0,583	0,610	58.2759 %
kmeans+SVM	0,676	0,333	0,675	0,676	0,674	0,685	67.5862 %
kmeans+ LogitBoost	0,634	0,364	0,637	0,634	0,635	0,633	63.4483 %
